# Ampelopsin Inhibits Cell Viability and Metastasis in Renal Cell Carcinoma by Negatively Regulating the PI3K/AKT Signaling Pathway

**DOI:** 10.1155/2021/4650566

**Published:** 2021-11-11

**Authors:** Zhonghe Zhao, Yan Jiang, Zhongguo Liu, Qingyan Li, Tiantian Gao, Shengxia Zhang

**Affiliations:** ^1^Department of Urology Surgery, Jiaozhou Central Hospital, Qingdao 266300, Shandong Province, China; ^2^Hemodialysis Room, The No. 4 People's Hospital of Jinan, Jinan 250031, Shandong Province, China; ^3^Department of Urology Surgery, Affiliated Qingdao Central Hospital, Qingdao University, Qingdao 266000, Shandong Province, China; ^4^Department of Clinical Laboratory, Zhangqiu District People's Hospital, Jinan 250200, Shandong Province, China; ^5^Department of Nephrology, Zhangqiu District People's Hospital, Jinan 250200, Shandong Province, China; ^6^Department of Nephrology Geriatrics, The Third People's Hospital of Qingdao, Qingdao 266041, Shandong Province, China

## Abstract

**Background:**

Previous studies have shown that Ampelopsin has an inhibitory effect on human tumors. However, the effect of Ampelopsin on renal cell carcinoma (RCC) is rarely reported. Therefore, this study aims to explain the role of Ampelopsin in RCC.

**Methods:**

Different concentrations of Ampelopsin (0, 10, 25, 50, and 100 *μ*M) were used to treat 786-O cells. Cell viability was detected by MTT assay, colony formation assay, and flow cytometry assay. Transwell assay and Wound healing assay were used to detect cell migration and invasion. Western blot analysis was applied to detect protein expression.

**Results:**

Ampelopsin inhibited cell proliferation and induced apoptosis in RCC. And Ampelopsin can inhibit cell migration and invasion in RCC. All these results changed in a dose-dependent manner. Ampelopsin (100 uM) had the strongest inhibitory effect on cell viability and metastasis. In addition, Ampelopsin negatively regulated the PI3K/AKT signaling pathway in RCC cells. Moreover, Ampelopsin was only cytotoxic to RCC cells.

**Conclusion:**

Ampelopsin inhibits cell viability and metastasis in RCC by negatively regulating the PI3K/AKT signaling pathway.

## 1. Introduction

Renal cell carcinoma (RCC) accounts for about 90% of renal malignancies, and the ratio of male to female is about 2 : 1. The age of onset was more than 40 years old, with a median age of 65 years [1]. RCC is usually a solid tumor; 10%–25% is cystic or mainly cystic cancer. The occurrence of RCC is related to smoking, occupation, obesity, genetics, and diabetes. Smoking is a high-risk factor for RCC [2]. For early localized RCC, surgical resection is the best treatment option. However, about 30% of patients with localized RCC will have local recurrence or distant metastasis after surgery [3]. Metastatic RCC has a poor prognosis and is not sensitive to radiotherapy and chemotherapy. The 5-year survival rate of metastatic RCC is less than 10% [4]. Moreover, there are no obvious symptoms in the early stage of RCC, and about 30% of patients are already metastatic RCC at the time of diagnosis [5]. Therefore, it is very important to find effective drugs for the treatment of metastatic RCC.

Ampelopsin is the main active ingredient of *Ampelopsis grossedentata*. *Ampelopsis grossedentata* has the effects of clearing away heat, detoxifying, and improving immunity [6]. Ampelopsin is mostly used in the adjuvant treatment of upper respiratory tract infection, fever, anti-inflammatory analgesia, swelling, and diuresis [7, 8]. Many studies have shown that Ampelopsin has the effect of inhibiting human tumors. For example, Ampelopsin induced apoptosis in human lung adenocarcinoma cells [9]. Ampelopsin inhibited the growth of breast cancer cell through the mitochondrial apoptosis pathway [10]. Ampelopsin reduced the migration and invasion of ovarian cancer cells by inhibiting epithelial-to-mesenchymal transition (EMT) [11]. However, the function of Ampelopsin in RCC is still unknown.

The PI3K/AKT signaling pathway plays an important role in a variety of biological processes such as cell metabolism, cell cycle, cell proliferation, and apoptosis [12]. Many traditional Chinese medicines play a role in human diseases by regulating the PI3K/AKT signaling pathway. For example, Ampelopsin inhibited the migration and invasion of human glioblastoma cells by regulating the PI3K/AKT signaling pathway [13]. The Chinese herbal formula tao hong si wu decoction protected cerebral ischemia-reperfusion injury through the PI3K/AKT signaling pathway [14]. In addition, it was also found that PI3K/AKT was involved in the pathogenesis of RCC. For example, HOXA6 inhibited cell proliferation and induced apoptosis by suppressing the PI3K/AKT signaling pathway in RCC [15]. Bufalin suppressed the proliferation and metastasis of RCC cells by inhibiting the PI3K/AKT/mTOR signaling pathway [16]. However, the interaction between Ampelopsin and PI3K/AKT signaling pathway has not been reported in RCC.

Therefore, the purpose of this study is to explore the role of Ampelopsin in RCC and explain the regulatory mechanism of Ampelopsin/PI3K/AKT signaling pathway in RCC.

## 2. Materials and Methods

### 2.1. Cell Culture

Normal kidney cells HK-2 and human RCC cells 786-O were purchased from Shanghai Institute of Biological Sciences (Shanghai, China). HK-2 cells were seeded in a 1 : 1 mixture of DMEM and Ham's F12 medium containing 10% fetal bovine serum (FBS, Life Technologies). 786-O cells were maintained in DMEM medium containing 10% FBS. They were cultured in a humidified incubator with 5% CO_2_ at 37°C.

### 2.2. Ampelopsin Treatment

Ampelopsin stock solution (0.1 M in dimethyl sulfoxide, HPLC ≥ 98%, Guilin Natural Ingredient, Inc., Guangxi, China) was added to the medium to reach the indicated concentrations (10, 25, 50, and 100 *μ*M). Then, different concentrations of Ampelopsin were incubated with the cells at 24 h and 48 h. A dimethyl sulfoxide solution without Ampelopsin was used as a blank control.

### 2.3. Determination of Cell Viability

To determine the cytotoxicity of Ampelopsin, MTT assay was performed to assess cell viability. 786-O and HK-2 cells (1 × 10^4^ cells/well) were seeded in 24-well plates with different concentrations of Ampelopsin and incubated at 37°C for 24 h or 48 h. After the exposure period, the cells were incubated with MTT (5 mg/mL) for 4 h. The 96-well plates were placed in the microplate reader to measure the absorbance at 540 nm.

### 2.4. Detection of Cell Proliferation

Human RCC cells 786-O were pretreated with different concentrations of Ampelopsin for 24 h. Next, they were seeded in 24-well plates (300 cells/well) and cultured in complete medium for 7 days. Then, the colonies were fixed with 10% formaldehyde for 10 min and stained with 1.0% crystal violet for 5 min. The number of colonies was counted.

### 2.5. Detection of Cell Apoptosis

The Annexin V/PI double staining method was used to detect the apoptosis rate. The RCC cell line 786-O (3 × 10^5^ cells/mL) was seeded in a 12-well plate. After 7 h, cells in each group were washed 3 times with PBS. Annexin V and PI solution (5 *μ*L, light-proof operation) were added and mixed. The cells were incubated for 20 minutes (surrounding avoidance). And apoptosis rate of each group was detected and analyzed by the flow cytometer.

### 2.6. Cell Migration

The RCC cell line 786-O (5 × 10^5^ cells/mL) was seeded into a 6-well plate and cultured in a 37°C, 5% CO_2_ incubator for 24 h. When the cell adhesion density reaches 80%–90%, a pipette was used to suck the cell liquid vertically and scrape it evenly. The cells were washed once with PBS to remove cell debris, and medium was added. After culturing in a 37°C, 5% CO_2_ incubator for 24 h, the cells were observed and photographed under an inverted microscope. Photoshop 7.0 software was used to analyze the migration distance of cells. And cell migration rate was calculated. Cell migration rate (%) = (0 h scratch spacing − 24h scratch spacing)/0 h scratch spacing × 100%.

### 2.7. Cell Invasion

Transwell chamber was placed at the bottom of the 24-well plate. 786-O cell suspension (1 × 10^6^ cells/mL) was added to the upper chamber. 600 *μ*L of medium was added to the lower chamber. The cells were cultured in a 37°C, 5% CO_2_ incubator for 24 h. After the culture is completed, the remaining cells on the inner surface of the upper membrane are wiped with a cotton swab. The invaded cells were fixed with 4% paraformaldehyde for 15 min and then stained with 0.1% crystal violet solution for 5 min. The invaded cells were observed and photographed under an inverted optical microscope.

### 2.8. Western Blot Assay

786-O cells were lysed with RIPA lysis buffer (BeyoTime Biotech) on ice to extract total protein. The BCA method was used for protein quantification. 40 *μ*g of total protein was separated by 12% SDS-PAGE and transferred to PVDF membranes (Millipore, USA). The membranes were then blocked with 5% skimmed milk and incubated with Bax, Bcl-2, E-cadherin, N-cadherin, vimentin, PI3K, p-PI3K, AKT, p-AKT, and GAPDH primary antibodies at 4°C overnight. Then, the secondary antibody was added and incubated at room temperature for 40 min. Chemiluminescence (ECL) was used for development. Quantity One 4.52 analysis software was used to measure the gray value of the band. Relative expression of target protein (IOD) = gray value of target protein/gray value of internal reference GAPDH.

### 2.9. Statistical Analysis

All experiments were repeated 3 times. SPSS 20.0 software was used for statistical analysis. The data are shown as mean ± SD. The statistically significant values were compared by Student's *t*-test or one-way ANOVA followed by Tukey's post hoc test. *P* < 0.05 is considered to indicate a statistically significant result.

## 3. Results

### 3.1. The Cytotoxicity of Ampelopsin to Normal Human Renal Cells and RCC Cells

**Figure 1:**
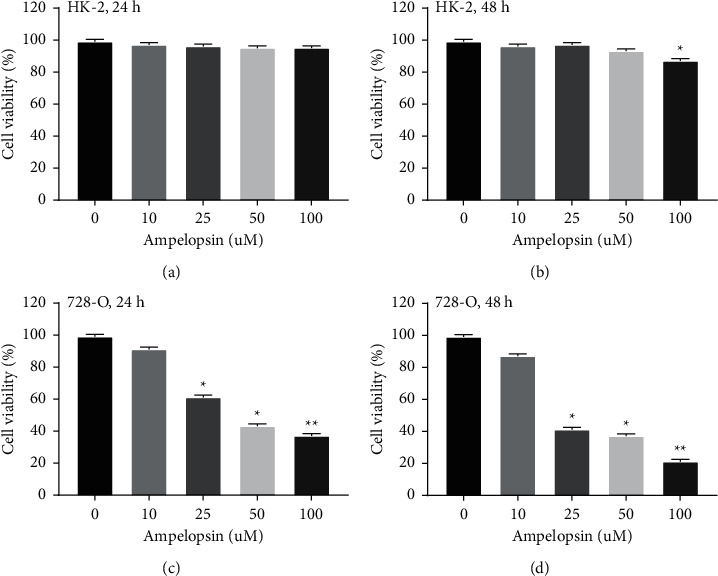
The cytotoxicity of Ampelopsin to normal human renal cells and RCC cells. (a- b) Normal kidney cell line HK-2 was treated with Ampelopsin (0, 10, 25, 50, and 100 uM) for 24 h and 48 h, and then the cell proliferation was detected by MTT analysis. (c-d) Human RCC cells 786-O were treated with Ampelopsin (0, 10, 25, 50, and 100 uM) for 24 h and 48 h, followed by MTT analysis to assess cell proliferation. ^*∗*^*P* < 0.05; ^*∗∗*^*P* < 0.01.

Normal kidney cells HK-2 and human RCC cells 786-O were treated with Ampelopsin at different concentrations (0, 10, 25, 50, and 100 uM) for 24 or 48 h. MTT assay showed that after 24 h of Ampelopsin treatment, no significant cytotoxicity was observed in normal kidney cells HK-2 (Figure 1(a)). We also found that 10, 25, and 50 uM Ampelopsin treatments were not cytotoxic to HK-2 cells at 48 h. However, 100 uM Ampelopsin had little effect on the survival of HK-2 cells for 48 h (*P* < 0.05; see Figure 1(b)). In addition, Ampelopsin (10, 25, 50, and 100 uM) inhibited the proliferation of 786-O cells in a dose-dependent manner for 24 h and 48 h (*P* < 0.05; see Figures 1(c) and 1(d)). These results indicate that Ampelopsin is only cytotoxic to RCC cells.

### 3.2. Ampelopsin Inhibits Cell Viability in RCC

**Figure 2:**
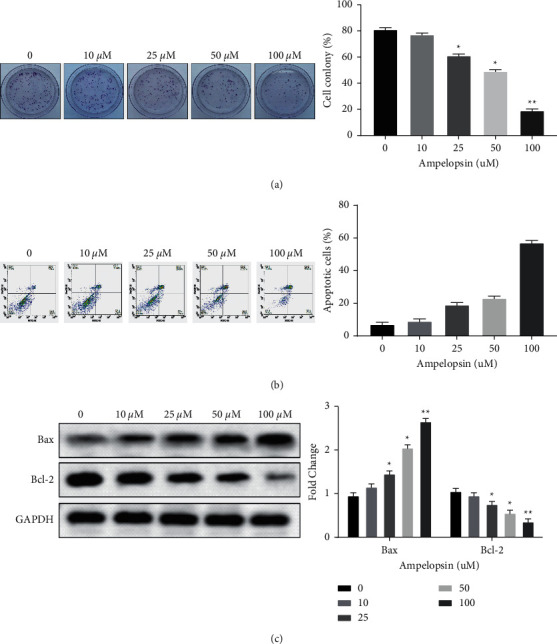
Ampelopsin inhibits cell viability in RCC. (a) The colony formation results of 786-O after treatments of Ampelopsin (0, 10, 25, 50, and 100 uM) for 24 h were exhibited. (b) Cell apoptosis was detected in 786-O cells treated with different concentrations of Ampelopsin (0, 10, 25, 50, and 100 uM). (c) The protein expression of Bax and Bcl-2 was measured in 786-O cells treated with different concentrations of Ampelopsin (0, 10, 25, 50, and 100 uM). ^*∗*^*P* < 0.05; ^*∗∗*^*P* < 0.01.

In order to further explore the effect of Ampelopsin on the viability of RCC cells, colony cell formation assay and flow cytometry analysis were performed. We found that Ampelopsin from 25 to 100 uM suppressed the proliferation of 786-O cells in a dose-dependent manner (*P* < 0.05; see Figure 2(a)). In addition, it was found that Ampelopsin (25 to 100 uM) induced a greater number of apoptotic cells in 786-O cells (*P* < 0.05; see Figure 2(b)). To further explore the underlying molecular mechanism, Western blot analysis was used to investigate the effect of Ampelopsin on Bax/Bcl-2 in 786-O cells. The results showed that Ampelopsin (25 to 100 uM) significantly reduced Bcl-2 and increased Bax levels in 786-O cells (*P* < 0.05; see Figure 2(c)). The above results demonstrate that Ampelopsin inhibits cell proliferation and induces apoptosis in RCC.

### 3.3. Ampelopsin Inhibits Cell Metastasis in RCC

**Figure 3:**
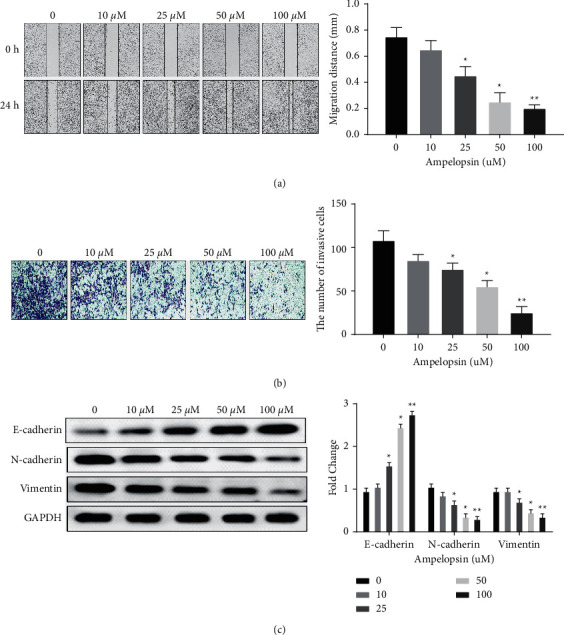
Ampelopsin inhibits cell metastasis in RCC. (a) Cell migration was detected by Wound healing assay in 786-O cells treated with different concentrations of Ampelopsin (0, 10, 25, 50, and 100 uM). (b) Cell invasion was detected by Transwell assay in 786-O cells treated with different concentrations of Ampelopsin (0, 10, 25, 50, and 100 uM). (c) The protein expression of E-cadherin, N-cadherin, and vimentin was measured in 786-O cells treated with different concentrations of Ampelopsin (0, 10, 25, 50, and 100 uM). ^*∗*^*P* < 0.05; ^*∗∗*^*P* < 0.01.

Next, the effect of Ampelopsin on cell metastasis was investigated in 786-O cells. Wound healing assay showed that the migration distance of 786-O cells treated with Ampelopsin (25 to 100 uM) was significantly shortened (*P* < 0.05; see Figure 3(a)), indicating that Ampelopsin can inhibit cell migration in RCC cells. Transwell assay showed that Ampelopsin (25 to 100 uM) significantly inhibited the invasion of 786-O cells (*P* < 0.05; see Figure 3(b)). In addition, the effect of Ampelopsin on EMT was investigated in 786-O cells. We found that Ampelopsin (25 to 100 uM) reduced the expression of N-cadherin and vimentin and enhanced E-cadherin expression in 786-O cells (*P* < 0.05; see Figure 3(c)). In summary, Ampelopsin can inhibit cell metastasis in RCC.

### 3.4. Ampelopsin Negatively Regulates the PI3K/AKT Signaling Pathway in RCC Cells

**Figure 4:**
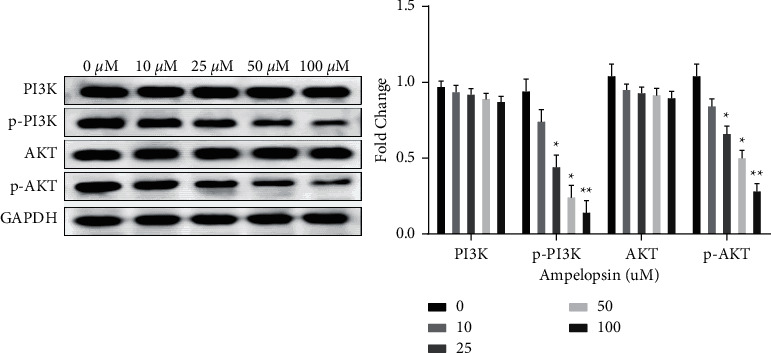
Ampelopsin negatively regulates the PI3K/AKT signaling pathway in RCC cells. The protein expression of PI3K, p-PI3K, AKT, and p-AKT was measured in 786-O cells treated with different concentrations of Ampelopsin (0, 10, 25, 50, and 100 uM). ^*∗*^*P* < 0.05; ^*∗∗*^*P* < 0.01.

In order to further clarify the possible underlying mechanism of Ampelopsin in RCC, the effect of Ampelopsin on the PI3K/AKT pathway was examined by Western blot analysis. The results showed a dose-dependent inhibition of p-PI3K and p-AKT in Ampelopsin-treated 786-O cells (*P* < 0.05; see Figure 4). However, the protein expression of PI3K and AKT in each drug group was not statistically different from that in the 0 uM group (*P* > 0.05; see Figure 4). Collectively, Ampelopsin negatively regulates the PI3K/AKT signaling pathway in RCC cells.

## 4. Discussion

RCC is resistant to almost all cellular drugs, and a small number of patients can be treated with high-dose interleukin-2 (IL-2), interferon, and other cytokines. In addition, RCC is not sensitive to radiotherapy and chemotherapy. Although the chemotherapy of RCC has been greatly improved, the prognosis of patients is still very poor. Many studies have shown that chemotherapy drugs isolated from natural plants can be effective killers of cancer cells. For example, Apigenin induced apoptosis by simultaneously suppressing Bcl-xl and Mcl-1 in colon cancer [17]. Curcumin inhibited cell proliferation and motility by suppression of TROP2 in bladder cancer [18]. In addition, the traditional Chinese medicine Bu-Shen-Jian-Pi-Fang has been reported to attenuate the glycolysis and immune escape of clear cell RCC [19]. Nevertheless, effective drugs are still needed to improve the prognosis of RCC patients.

Ampelopsin is a naturally occurring flavonoid compound found in grapes, fruits, vegetables, and herbs [20]. Ampelopsin has attracted the attention of researchers because of its biological activities such as antifatigue, anti-inflammatory, antitumor, liver protection, and regulation of lipid metabolism [21, 22]. More importantly, Ampelopsin is almost nontoxic to animal models [23]. In this study, we also found that Ampelopsin had no significant cytotoxicity to normal human kidney cells. In addition, it was found that Ampelopsin can inhibit cell viability and metastasis in RCC cells. Similar to our results, Ampelopsin has been reported to inhibit cell proliferation and induce apoptosis in leukemia by downregulating the AKT signaling pathway [24]. Ampelopsin reduced the migration and invasion of ovarian cancer cells by inhibiting EMT [11]. These results demonstrate that Ampelopsin has an antitumor effect in RCC.

**Figure 5:**
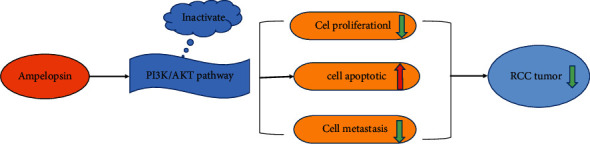
Ampelopsin inhibits cell viability and metastasis in RCC by negatively regulating the PI3K/AKT signaling pathway.

In the current study, Ampelopsin suppressed cell metastasis in RCC by inhibiting EMT. EMT is related to the acquisition of invasion and migration characteristics and provides tumor cells with the ability to invade adjacent tissues [25]. Here, the expression of E-cadherin in 786-O cells was increased in a dose-dependent manner by Ampelopsin, while N-cadherin and vimentin were decreased. This indicates that EMT is inhibited by Ampelopsin. Therefore, we infer that Ampelopsin at least partly reduces the metastasis of RCC cells to prevent the development of RCC. In addition, we found that Ampelopsin negatively regulated the PI3K/AKT signaling pathway in RCC cells. Qi et al. reported that Ampelopsin reduced endotoxin inflammation by inhibiting the activation of PI3K/AKT/NF-kappaB signaling pathway [26]. The above result suggests that Ampelopsin may inhibit the progression of RCC by blocking EMT and PI3K/AKT pathways (Figure 5). However, our conclusion has not been verified by *in vivo* experiment. Thus, animal experiment will be performed in the future to further confirm our results.

## 5. Conclusion

In summary, Ampelopsin can inhibit cell viability and metastasis in RCC in a dose-dependent manner. Ampelopsin may play an antitumor effect in RCC by negatively regulating the PI3K/AKT signaling pathway. In addition, Ampelopsin has low cytotoxicity to normal human kidney cells. Therefore, Ampelopsin can be considered as a promising strategy to prevent the development of human RCC.

## Data Availability

The datasets used during the present study are available from the corresponding author upon reasonable request.
